# High-Efficiency Biocidal Solution Based on Radiochemically Synthesized Cu-Au Alloy Nanoparticles

**DOI:** 10.3390/nano11123388

**Published:** 2021-12-14

**Authors:** Eduard-Marius Lungulescu, Radu Setnescu, Eros A. Pătroi, Magdalena V. Lungu, Delia Pătroi, Ioana Ion, Radu-Claudiu Fierăscu, Raluca Șomoghi, Miruna Stan, Nicoleta-Oana Nicula

**Affiliations:** 1 National Institute for Research and Development in Electrical Engineering ICPE-CA, 313 Splaiul Unirii, 030138 Bucharest, Romania; radu.setnescu@icpe-ca.ro (R.S.); eros.patroi@icpe-ca.ro (E.A.P.); magdalena.lungu@icpe-ca.ro (M.V.L.); delia.patroi@icpe-ca.ro (D.P.); ioana.ion@icpe-ca.ro (I.I.); nicoleta.nicula@icpe-ca.ro (N.-O.N.); 2 Department of Advanced Technologies, Faculty of Sciences and Arts, Valahia University of Târgoviște, 13 Aleea Sinaia, 130004 Targoviste, Romania; 3 National Institute for Research and Development in Chemistry and Petrochemistry—ICECHIM Bucharest, 202 Spl. Independentei, 060021 Bucharest, Romania; radu_claudiu_fierascu@yahoo.com (R.-C.F.); r.somoghi@gmail.com (R.Ș.); 4 Department of Biochemistry and Molecular Biology, Faculty of Biology, University of Bucharest, 91-95 Splaiul Independentei, 050095 Bucharest, Romania; miruna_stan@yahoo.com; 5 Research Institute of the University of Bucharest—ICUB, University of Bucharest, 050657 Bucharest, Romania

**Keywords:** radiochemical synthesis, Cu-Au NPs, biocidal efficiency, surface decontamination, medical areas

## Abstract

The use of nanotechnologies in the applied biomedical sciences can offer a new way to treat infections and disinfect surfaces, materials, and products contaminated with various types of viruses, bacteria, and fungi. The Cu-Au nanoparticles (NPs) were obtained by an eco-friendly method that allowed the obtaining in a one-step process of size controlled, well dispersed, fully reduced, highly stable NPs at very mild conditions, using high energy ionizing radiations. The gamma irradiation was performed in an aqueous system of Cu^2+^/Au^3+^/Sodium Dodecyl Sulfate (SDS)/Ethylene Glycol. After irradiation, the change of color to ruby-red was the first indicator for the formation of NPs. Moreover, the UV-Vis spectra showed a maximum absorption peak between 524 and 540 nm, depending on the copper amount. The Cu-Au NPs presented nearly spherical shapes, sizes between 20 and 90 nm, and a zeta potential of about −44 mV indicating a good electrostatic stability. The biocidal properties performed according to various standards applied in the medical area, in dirty conditions, showed a 5 lg reduction for *Staphylococcus aureus*, *Pseudomonas aeruginosa*, and *Enterococcus hirae*, a 5 lg reduction for both enveloped and non-enveloped viruses such as Adenovirus type 5, Murine Norovirus, and human Coronavirus 229E, and a 4 lg reduction for *Candida albicans*, respectively. Thus, the radiochemically synthesized Cu-Au alloy NPs proved to have high biocide efficiency against the tested bacteria, fungi, and viruses (both encapsulated and non-encapsulated). Therefore, these nanoparticle solutions are suitable to be used as disinfectants in the decontamination of hospital surfaces or public areas characterized by high levels of microbiological contamination.

## 1. Introduction

In recent years, amid the global health crisis caused by the Sars CoV-2 virus, research has been intensified to obtain effective solutions to fight against infections caused by various microorganisms, especially in professional environments (e.g., hospitals, medical clinics, schools). According to some studies [1,2], around 90% of intra-hospital infections (of bacterial and viral types) are spread by contaminated surfaces, such as medical items, protection equipment, tables, beds, doors, switches, pillows, and mattresses, due to their intrinsic porous structure [3] a.s.o. In addition, the prevention and control of infections associated with medical treatments (healthcare-associated diseases) are priority concerns for health systems at a global scale. These microorganisms can cause various infections (affecting respiratory, gastrointestinal or urinary tracts, blood, or ENT system [4]) that are challenging to treat, most of them being antibiotic-resistant [3]. Their incidence rate is 5–10% of hospitalized patients. In addition, it was shown that, due to the high viability of the SARS CoV-2 virus in aerosols and on various contaminated surfaces, medical staff members have been contaminated even if rigorous protection measures were taken [2,5].

Nowadays, new NPs-based technologies are continuously introduced and developed in many application fields, including biomedical and pharmaceutic products, which are very dynamic domains.

Metal alloy nanoparticle systems have unique physical and chemical properties enabling them to be used in various possible applications such as sensors, renewable energy technologies, catalysts, medical imaging, or antimicrobial agents. Essential parameters in tuning their properties are particle size, particle distribution, and shape [6].

As in the case of the synthesis of monometallic NPs, the morphology and crystal structure of metal alloy NPs can be controlled in particular by choosing appropriate methods of synthesis and control of some reaction parameters, such as the concentration of precursor ions and reducing agents, stabilizing agents, temperature, and time [6,7]. Thus, the metal alloy NPs can be obtained using both top-down and bottom-up approaches [8] by different methods, such as: chemical (e.g., chemical and electrochemical reduction [9], hydrothermal [10], precipitation [11], sol-gel [12], micro-emulsion [13]), physical (e.g., sputtering [14], thermal decomposition [15], microwaves [16], radiolytic [17], and sonochemical [18]) and biological (bacteria, fungi, plants, agricultural and industrial wastes) [19,20,21,22] procedures. Metal alloy NPs have an advantage over monometallic nanoparticle systems due to the synergism between the individual component alloy nanoparticle’s characteristics and a more stable structure [8].

Frequently, the synthesis methods are expensive, requiring special conditions, long processing time, and involving the use of chemical reduction agents, which would be harmful to the environment and would present enhanced biologic risks for humans [23]. Unlike conventional synthesis methods, the synthesis based on the chemical effect of gamma rays is a particularly ecologic and rapid method. It leads to high purity metal NPs with controllable dimensions and shape, in large amounts, depending only on the available irradiation volume, enabling the development of industrial-scale processes. This method does not require any chemical or biological reducing agents, avoiding any risks related to their toxicity or bio-toxicity [7,24,25].

Recently, research was conducted to develop different Au-based metal alloy nanoparticle systems (e.g., Au-Ag, Au-Pd, Au-Pt) [6], among which Cu-Au-based systems have aroused high interest for various applications in catalysis (CO_2_ and p-nitrophenol reduction, methanol, and CO oxidation [6]), photonics (sensors for the detection of persistent toxic organic pollutants [26]), or biomedical research (photothermal therapy [27]). The alloying of copper with gold is an effective method to reduce the gold costs and to improve the stability of copper, while benefiting from their individual properties: Au is a biocompatible element with high stability, size-tunable surface plasmon resonance, fluorescence, and easy-surface functionalization [28], and copper has unique catalytic and optical properties [29,30,31].

Although both elements have remarkable antimicrobial properties, as shown in numerous studies [29,32,33,34,35], to our best knowledge, we have not found any study to discuss the antimicrobial activity of Cu-Au NPs. The biocide activity of metal NPs is well known and occurs with different intensities depending on the synthesis procedure, concentration, dimensions, and the shape of the NPs, but their action mechanism is not fully understood [24,36,37]. However, several general mechanisms of the antimicrobial action of NPs are accepted in this case, such as the attachment of NPs to the cell membrane and inhibition of binding to the ribosomes of transfer ribonucleic acid molecules (tRNA), generation of reactive oxygens species (ROS) on the particle surface, diffusion and drilling of cell membranes, prevention of the biofilm formation, etc. [34,35,37].

The aim of this study was to synthetize the bimetallic NPs of Cu-Au alloy via a gamma radiation procedure. The resulting NPs were characterized in terms of optical, morpho-structural, and biocidal properties. The biocidal activity was evaluated from both a qualitative and quantitative point of view, according to test standards applicable to the medical purposes. These nanoparticle systems proved to have high biocide efficiency against bacteria, fungi, and viruses (both encapsulated and non-encapsulated); hence, they are suitable for use as surface disinfectants in highly contaminated environments.

## 2. Materials and Methods

### 2.1. Chemicals

Copper (II) sulphate pentahydrate (Mw = 249.69 g/mol, ≥98%, Sigma Aldrich, St. Louis, MO, USA) and gold (III) chloride hydrate (Mw = 339.79 g/mol, p.a., ≥49%Au, Fluka Analytical, Munich, Germany) were used as precursor salts for Cu-Au NPs; dodecyl sulphate sodium salt (SDS, Mw = 288.38 g/mol, Ph Eur, Merck, Darmstadt, Germany) and ethylene glycol (EG, Mw = 62.07 g/mol, Chimreactiv SRL, Bucharest, Romania) were used as a capping agent and for scavenging of hydroxyl radicals, respectively. These substances were used in the as-received form, without further purifications.

Ultrapure water (18.6 MΩ produced by Simplicity UV Water Purification System, Milli-Q, Merck, Darmstadt, Germany) was used as a solvent.

### 2.2. Preparation of Cu-Au NPs

Cu-Au NPs were obtained by a radiolytic synthesis method: an aqueous solution of SDS (0.8% *w/v*), used as a stabilizing agent, Cu^2+^ and Au^3+^ salts, corresponding to a concentration of 1 mM each, were dissolved under magnetic stirring (800 rpm) at room temperature (Lungulescu et al., 2020). Different concentrations of copper ions were used (0%, 50%, 75%, and 90%) and the balance gold ions to obtain Au NPs, Cu-Au (1:1) NPs, Cu-Au (3:1) NPs, and Cu-Au (9:1) NPs. An amount of ethylene glycol (15% *v/v*) was added to this solution to increase the efficiency of both the reduction process and HO radicals trapping (generated in the water radiolysis process). The solution was bubbled with argon for 30 min for deaeration, at a low gas flow rate (50 mL/min), and subsequently it was sealed in an airtight container and subjected to irradiation. Gamma irradiation was performed in a laboratory irradiation facility (Ob-Servo Sanguis, Izotop, Budapest, Hungary), at room temperature and a dose rate of 0.7 kGy/h; the integrated irradiation dose was 30 kGy.

### 2.3. Characterization of Cu-Au NPs

#### 2.3.1. UV-Vis

UV-Vis absorption spectroscopy (V-570 UV-Vis spectrophotometer, Jasco, Tokyo, Japan) was used to determine the optical properties, to confirm the formation of Cu-Au NPs, and to study their stability after 1 year. The analyses were performed in the wavelength range of 450–700 nm, using quartz cells with a 1 cm optical path, at a resolution of 1 nm. The reference was an SDS/EG solution, irradiated under similar conditions.

#### 2.3.2. FTIR

To confirm the SDS coating/stabilization capacity of Cu-Au NPs, infrared spectroscopy (FTIR-4200 spectrometer, Jasco, Tokyo, Japan) was used. The colloidal nanoparticle solutions and the initial polymer solution were spread in a thin layer on a ZnSe window and measured in transmission mode in the spectral range of 4000–500 cm^−1^, at a resolution of 1 cm^−1^, and 200 scans for each spectrum.

#### 2.3.3. XRD

For the X-ray diffraction (XRD) analysis, the liquid samples of the Cu-Au NPs/SDS/EG system were pipetted onto a “zero-background” (911) cut Silicon sample holder (Siltronix, Armchamps, France) and exposed for 24 h at room temperature until the liquid medium evaporated. In addition, the SDS/EG system was investigated in its crystalline solid form to evaluate its specific angular position in the experimental patterns of the Cu-Au/SDS/EG Np system. Their analysis was performed using a D8 Discover diffractometer (Bruker, Billerica, MA, USA) configured in theta-2theta geometry, having on primary optics a Cu radiation (λKα1 = 1.540598 Å) X-ray anode tube and a 0.6 mm Göebel mirror, and on secondary optics a 1D LynxEye detector. XRD patterns were recorded in an angular increment of 0.04° at a scan rate of 10 s/step, then were indexed using the ICDD PDF 2 database.

#### 2.3.4. TEM

The morphology of the Cu-Au NPs was examined using transmission electron microscopy (TECNAI G2 F20 TWIN Cryo-TEM, FEI Company, Hillsboro, OR, USA) with an accelerating voltage of 200 keV. A small droplet from both dispersions of Cu-Au (1:1) NPs (just after irradiation and after 1 year) was placed on copper grids with Lacey carbon film, without staining.

#### 2.3.5. DLS and ELS

The hydrodynamic diameter (effective diameter) of Cu-Au NPs and particle size distribution were determined by a dynamic light scattering (DLS) technique known also as photon correlation spectroscopy (PCS), which measures the fluctuations in light scattering intensity due to random Brownian motion of the particles, using a 90Plus nanoparticle size analyzer (Brookhaven Instruments Corporation, Holtsville, NY, USA) equipped with a solid-state laser with 660 nm wavelength and an output power of 32 kW. The light scattering was performed at a scattering angle of 90° and a temperature of 25 °C using a 10 mm pathlength plastic cell filled with 3 mL of unfiltered Cu-Au NP solution.

The dispersion stability was investigated by zeta potential measurements determined by the electrophoretic light scattering (ELS) method using the 90Plus apparatus (Brookhaven Instruments Corporation, Holtsville, NY, USA) with the BI-Zeta option having a dip-in electrode system of AQ-622 type. The zeta potential was determined by measuring the electrophoretic mobility of the surface charged Cu-Au NPs dispersed in an aqueous solution with a viscosity of 0.89 cP, a refractive index of 1.33, and a dielectric constant of 78.54, under a current of 18.30–19.44 mA corresponding to an electric field of 15.37–15.78 V/cm. The electrophoretic mobility measurements were performed at a temperature of 25 °C in the same cells filled with Cu-Au NPs solution as used in the DLS measurements having a pH of 2.8. The conductance of Cu-Au NPs solution was determined also during the ELS measurements. Each reported value was an average of 10 measurements.

#### 2.3.6. Biocidal Activity

##### Qualitative Assessments of Antimicrobial Activity

The qualitative antimicrobial assessment of colloidal Cu-Au NPs was performed by using an adapted diffusion disk method (CLSI, 2020). From each tested strain, *Escherichia coli* ATCC 25922, *Staphyloccocus aureus* ATCC 9737, a bacterial suspension with a cellular density of 0.5 McFarland (1.5 × 10^8^ CFU/mL), was prepared in sterilized distilled water. Then, 10 µL of each Cu-Au NP solution was seeded on the Mueller Hinton (MH) agar medium, previously inoculated with the as-prepared bacterial suspension, and incubated for 24 h at 37 °C. The antibacterial efficiency was evaluated by measuring the diameter of the growth inhibition zone (DGIZ) with a ruler. To have an image on the influence of some dilutions on NPs’ bactericidal efficiency, diluted solutions in concentrations of 50%, 25%, and 12.5% were also tested.

##### Quantitative Assessment of Biocidal Activity

To simulate the real operating conditions, the antibacterial activity of Cu-Au NPs was tested on a laboratory surface [38] and compared with a solution of 70% ethanol. Briefly, two ceramic surfaces, previously cleaned with 70% ethanol solution (to eliminate any potential background contamination), were contaminated, separately, with 1 mL of *Escherichia coli* ATCC 25922 and *Staphyloccocus aureus* ATCC 9737, respectively (both with a cellular density of 0.5 McFarland (1.5 × 10^8^ CFU/mL). Each contaminated surface was divided into three squares of 25 cm^2^. The first square was treated with 1 mL of 70% ethanol solution, the second one with 1 mL of Cu-Au (1:1) NPs, and the third one was kept untreated (i.e., only inoculated with bacteria). After a contact time of 1 h, the antibacterial efficiency was highlighted by collecting samples using a sterile applicator moistened in distilled water and their seeding on a MH agar medium for 24 h at 37 °C. After the incubation time, the number of colonies/25 cm^2^ was counted.

##### Evaluation of the Bactericidal Activity

A bactericidal efficacy test was performed according to EN 13727:2016 [39] on three bacterial strains (*Staphylococcus aureus* ATCC 6538, *Pseudomonas aeruginosa* ATCC 15442, and *Enterococcus hirae* ATCC 10541) for a 60 min contact duration at a temperature of 20 ± 1 °C. The Cu-Au NPs solution was tested without any dilution. The most restrictive assay condition regarding interfering substances was selected. Therefore, dirty conditions (recommended for application without previous cleaning) were represented by a mixture of 3 g/L bovine serum albumin (Sigma Aldrich, St. Louis, MO, USA) and 3 mL/L of erythrocytes (BioMerieux, Craponne, France) as indicated in the standard. The contact between microorganisms and the tested solution was stopped by adding a neutralization fluid (composed of 1 g/L peptone, 3 g/L lecithin, 1 g/L L-histidine chloride, 3.6 g/L monopotassium phosphate, 7.2 g/L disodium phosphate, 4.3 g/L sodium chloride, and 30 mL/L polysorbate 80) as indicated in the standard. Furthermore, the absence of any toxic effect induced by the experimental conditions and neutralizer was confirmed according to the standard.

After neutralization, the viable bacteria were counted after incubation at 36 ± 1 °C in the corresponding agar for the control and the tested suspension. The assay was performed in duplicate, and the calculations were made by subtracting the logarithmic values of the control and test results. The bactericidal activity was considered efficient against the microorganisms for a decimal log (lg) reduction value equal to or higher than 5.

##### Evaluation of the Fungicidal Activity

A fungicidal efficacy assay was performed according to EN 13624:2014 [40] on two strains (*Candida albicans* ATCC 10231 and *Aspergillus brasiliensis* ATCC 16404) for a 60 min contact duration at a temperature of 20 ± 1 °C in dirty conditions represented by a mixture of 3 g/L bovine serum albumin and 3 mL/L of erythrocytes. The Cu-Au NPs solution was tested as pure (80%). The method involved the dilution-neutralization assay, with the neutralizer being a mixture of 5 g/L tryptone, 2.5 g/L yeast extract, 10 g/L dextrose, 1 g/L sodium thioglycolate, 1 g/L sodium thiosulfate, 2.5 g/L sodium bisulphite, 7 g/L soya lecithin, 5 g/L polysorbate 80, 1 g/L glycine, 1 g/L L-histidine, and 30 g/L saponin. After neutralization, the viable fungi were counted after incubation at 30 ± 1 °C in the corresponding medium for the control and the tested suspension. The assay was performed in duplicate and calculations were made by subtracting the logarithmic values of the control and test results. The material was considered efficient against the microorganisms if the decimal log (lg) reduction value was equal to or higher than 4.

##### Evaluation of the Virucidal Activity

The virucidal efficacy of the tested solution was evaluated according to EN 14476:2013+A2:2019 [41] on four strains (Poliovirus type 1 ATCC VR-192, Adenovirus 5 ATCC VR-5, Murine Norovirus strain S99 Berlin, and Coronavirus 229E ATCC VR-740) for a 60 min contact duration at a temperature of 20 ± 1 °C in dirty conditions, represented by a mixture of 3 g/L bovine serum albumin and 3 mL/L of erythrocytes. The Cu-Au NPs solution was tested at two different concentrations (pure—80% and 50%). The method involved the dilution-neutralization assay. Upon completion of the contact time, the reaction mixtures were immediately neutralized by adding the ice-cold neutralizer (a mixture of 5 g/L tryptone, 2.5 g/L yeast extract, 10 g/L dextrose, 1 g/L sodium thioglycolate, 1 g/L sodium thiosulfate, 2.5 g/L sodium bisulphite, 7 g/L soya lecithin, 5 g/L polysorbate 80, 1 g/L glycine, 1 g/L L-histidine, and 30 g/L saponin) to stop the virucidal reaction. Neutralized test samples were further serially ten-fold diluted in a dilution medium and inoculated onto host cells to assay for the infectious virus using a 50% tissue culture infectious dose (TCID_50_) assay. The results were expressed in units of a decimal log (lg) TCID_50_ per mL (TCID_50_/mL). The material was considered efficient against the viruses if the decimal log (lg) reduction value was equal to or higher than 4.

#### 2.3.7. Evaluation of Human Cytotoxicity

Human skin fibroblasts CCD-1070Sk were grown in a minimum essential medium containing 10% fetal bovine serum (Gibco, Waltham, MA, USA) at 37 °C in a humidified atmosphere with 5% CO_2_. The cells were seeded at a cell density of 10^5^ cells/cm^2^ and left to adhere for 24 h. Then, the fibroblasts were incubated for the next 6 h with different dilutions (1/50, 1/100, 1/200, 1/500, 1/1000, and 1/2000) of a tested sample, which was previously sterilized by exposure to ultraviolet light for one hour. Untreated cells were used as a control for all in vitro experiments.

##### MTT Assay

The cellular viability was measured using the 3-(4,5-dimethylthiazol-2-yl)-2,5-diphenyltetrazolium bromide (MTT; Sigma Aldrich, St. Louis, MO, USA) assay. After 24 h of incubation, the culture medium was removed, and the cells were incubated with 1 mg/mL MTT for 2 h at 37 °C. The purple formazan crystals formed in the viable cells were dissolved with 2-propanol (Sigma-Aldrich, St. Louis, MO, USA) and the absorbance was measured at 595 nm using a microplate reader (Flex Station, Molecular Devices, San Jose, CA, USA).

##### Griess Assay

The concentration of nitric oxide (NO) in the collected culture medium after the 24 h of incubation was performed with the Griess reagent, a stoichiometric solution (*v/v*) of 0.1% naphthylethylendiamine dihydrochloride and 1% sulphanilamide in 5% H_3_PO_4_). Increased levels of NO are related to cytotoxic effects as this molecule is connected with inflammation and apoptosis. The absorbance of the mix formed by equal volumes of the medium supernatants and the Griess reagent was read at 550 nm using the FlexStation 3 microplate reader. The NO concentration was calculated from the NaNO_2_ standard curve.

##### Statistical Analysis

The in vitro assays were performed in triplicates and the results were presented as mean ± standard deviation (SD) of three independent experiments. The statistical significance was analyzed by Student’s *t*-test. A value of *p* less than 0.05 was considered significant.

## 3. Results and Discussions

### 3.1. Radiochemical Synthesis of Cu-Au NPs

The radiochemical synthesis of metal NPs (monometallic or alloy) has several advantages compared to conventional synthesis methods: it is simple, rapid, and can be achieved in mild conditions such as ambient pressure and temperature and with a high degree of reproducibility [42]. In addition, the reduction of metal ions to zero-valent metal atoms is performed without chemical or biological reducing agents and allows for obtaining large quantities of NPs with uniform dispersion and high stability, controllable size and structure, and at low costs (if an irradiation facility is already available). Hence, this method is suitable for application at an industrial scale [7,25,43]. The control of the nanoparticle cluster size and the crystal structure is achieved by controlling a few synthesis parameters, of which the most important is the irradiation dose [24].

The radiochemical synthesis of metallic NPs is based on water radiolysis induced by gamma radiation. This process generates different radiolytic species [44] (Equation (1)) that have strong reducing properties, such as *hydrated electron* ($e_{aq}^{-}$) and *hydrogen atoms* (H^●^), which have a redox potential of −2.87 V_NHE_ and −2.30 V_NHE_, respectively [45]. These species can reduce the metal ions present in the solution to their zero-valence state, followed by their agglomerations until stable NPs are obtained.

(1) $$H_{2}O\;\overset{\gamma }{\to }\;e_{aq}^{-},\;H_{3}O^{+},\;H•,\;H_{2},\;\mathrm{OH}•,\;H_{2}O_{2}$$

In addition, the water radiolysis produces hydroxyl radicals (OH^●^). These oxidizing species have a redox potential of +2.8 V_NHE_, and could oxidate the metal NPs [45]; therefore, their annihilation is mandatory. In the present work, Cu-Au alloy NPs are synthesized by irradiating an aqueous solution containing Cu and Au ions, in the presence of SDS used as a stabilizing agent and ethylene glycol used both as hydroxyl radical scavenging agent and as an aid to increase the efficiency of reducing metal ions.

During gamma irradiation, the Cu and Au ions are reduced by reducing species generated by water radiolysis:

(2) $${\mathrm{Cu}}^{2+}+2e_{aq}^{-}\to {\mathrm{Cu}}^{0}\;{\mathrm{Cu}}^{2+}+2H●\to {\mathrm{Cu}}^{0}+H_{2}$$

(3) $${\mathrm{Au}}^{3+}+3e_{aq}^{-}\to {\mathrm{Au}}^{0}\;{\mathrm{Au}}^{3+}+6H●\to {\mathrm{Au}}^{0}+3H_{2}$$

**Figure 1:**
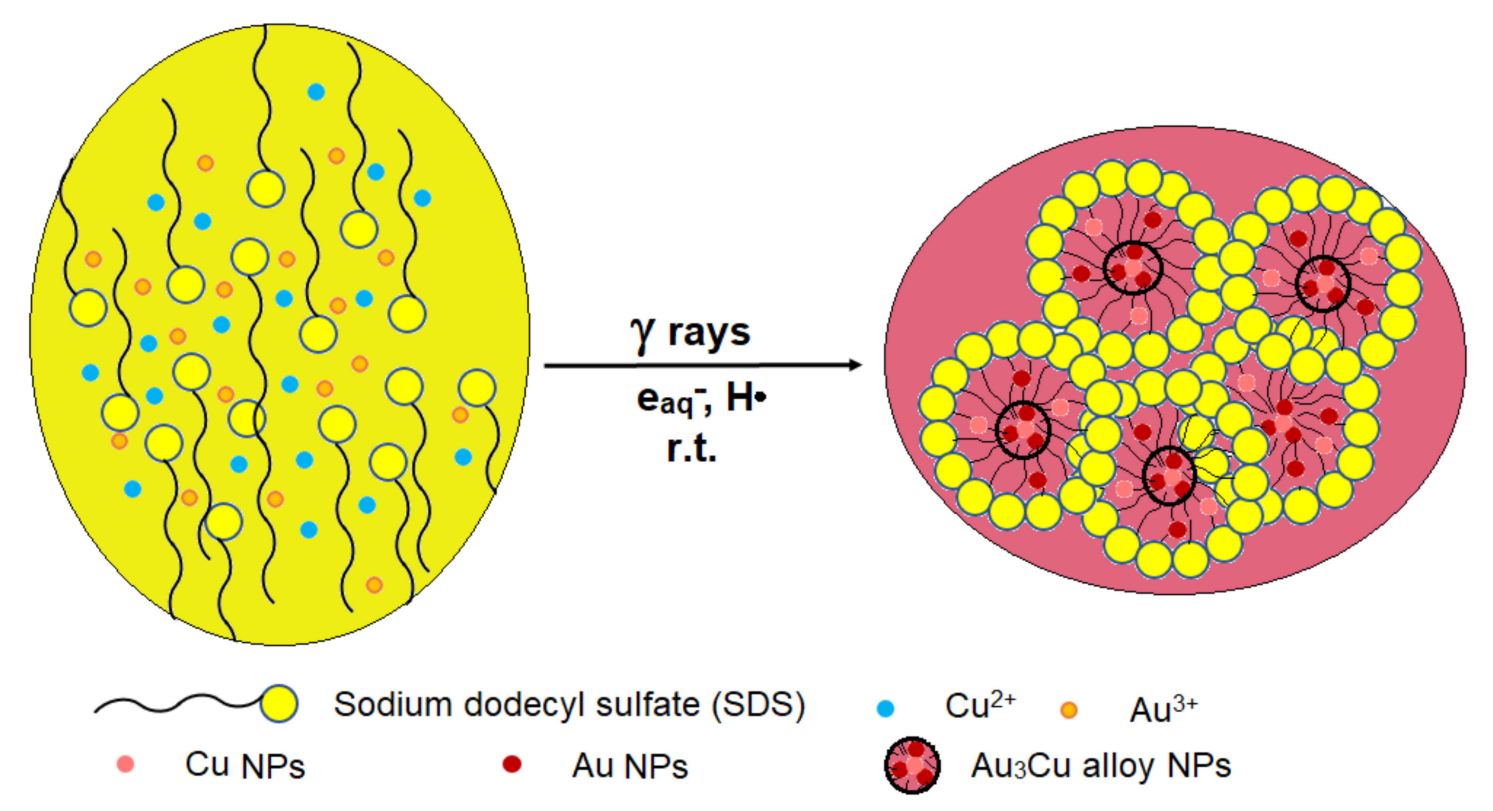
Radiochemical synthesis of Cu-Au NPs and role of SDS.

Gold ions are reduced faster than copper ions, due to their different reduction potentials of Cu^2+^/Cu^0^ (i.e., +0.34 V) and Au^3+^/Au^0^ (i.e., +1.5 V) [46]. The addition of SDS contributes to the stabilization of the alloy NPs by powerful electrostatic interactions (as shown from FTIR studies) between the hydrophobic structure of the alkyl chain of SDS molecules and the surface of the alloy NPs [47] (Figure 1).

**Scheme 1:**
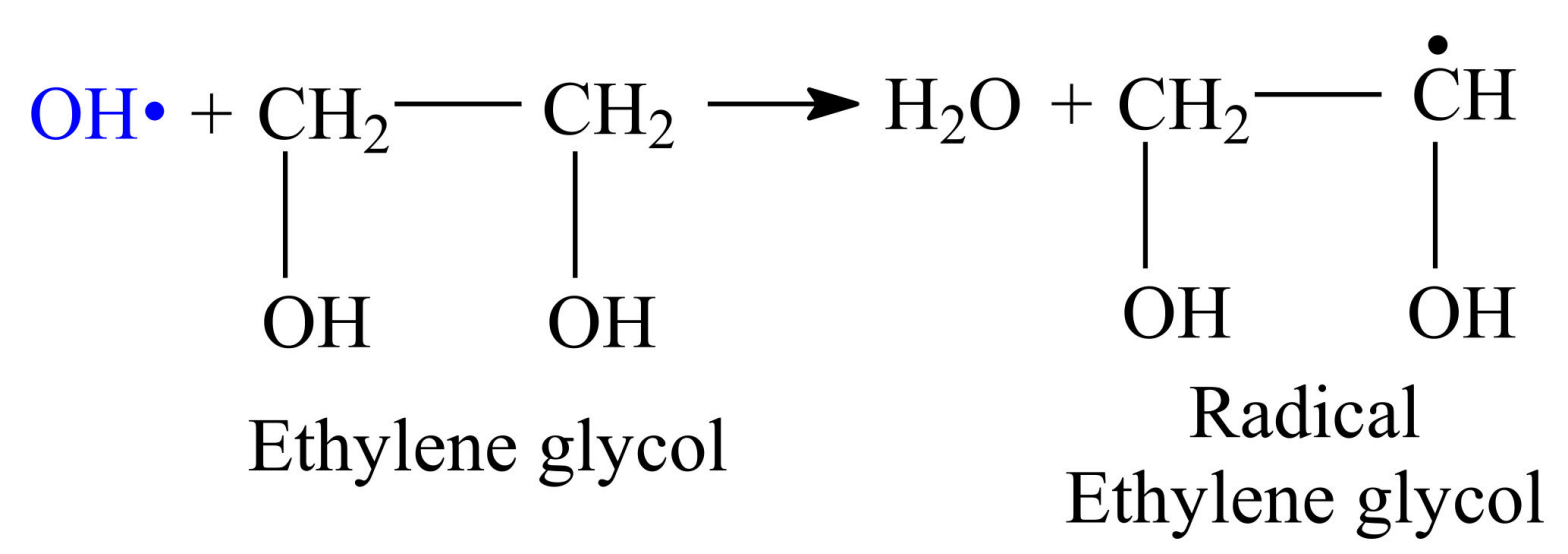
The mechanism of hydroxyl radicals scavenging by EG.

To prevent the oxidation of Cu-Au alloy NPs, ethylene glycol is added to scavenge the hydroxyl radicals (Scheme 1).

**Scheme 2:**
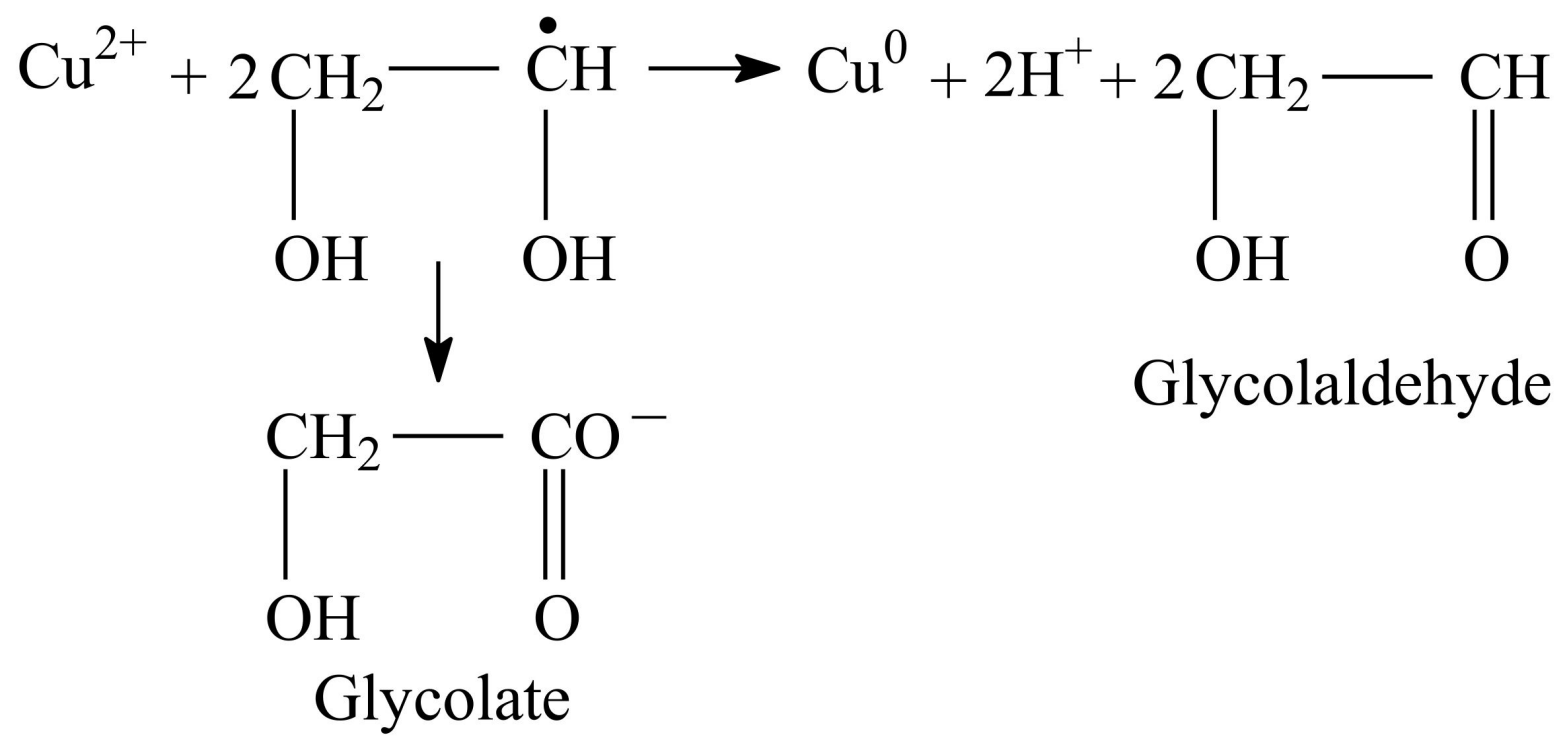
The mechanism of Cu^2+^ reduction by EG radicals.

According to Kugai et al. [17], the radical ethylene glycol could reduce metal ions forming glycolaldehydes or glycolates (Scheme 2). The glycolate is a good stabilizer and can be chemisorbed on the metal surface to suppress crystal growth and promote copper alloying with gold [17].

**Figure 2:**
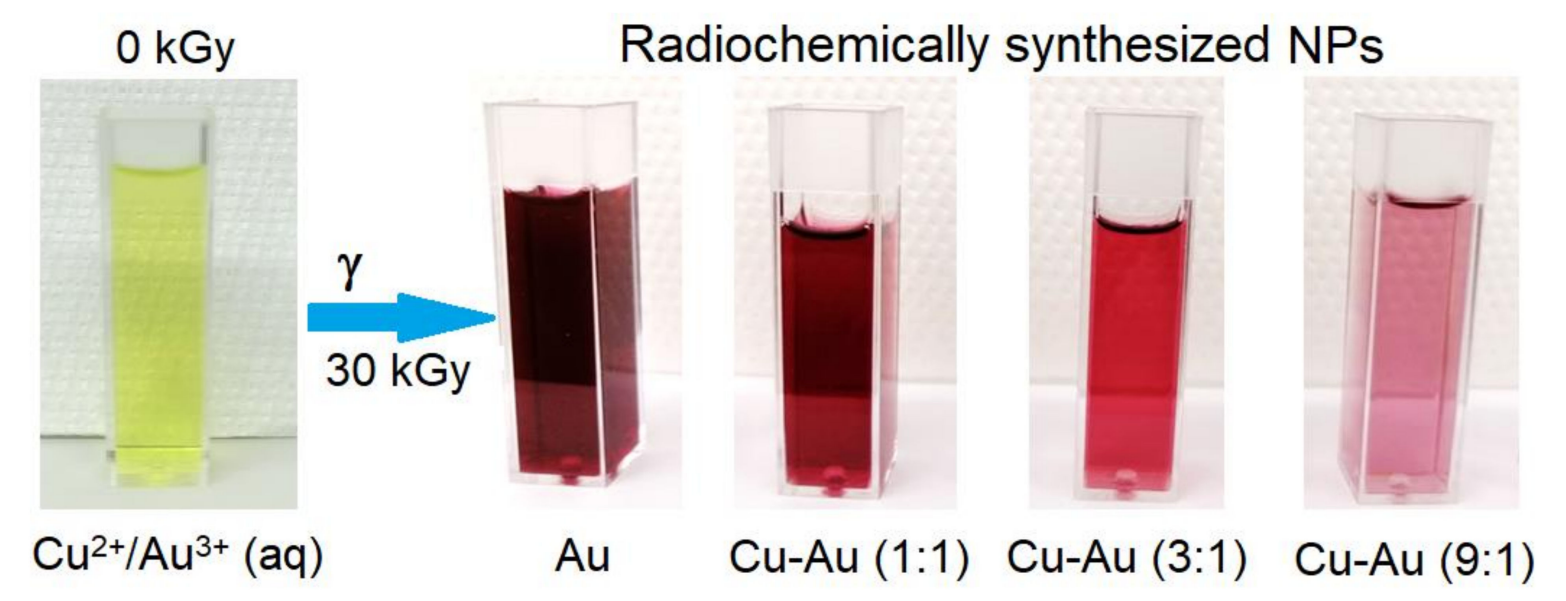
Color change of the solution induced by irradiation (30 kGy) and Cu-Au ions concentration.

During gamma irradiation, the solution of SDS and ethylene glycol, containing dissolved Au and Cu salts, in previously established concentration ratios, changes color from yellow-green to ruby-red, due to the formation of Cu and Au-based NPs (Figure 2). The increasing copper ions concentration in the initial solution leads to the color intensity changes of irradiated solutions from ruby-red (for Cu-Au (1:1) NPs) to pale pink (for Cu-Au (9:1) NPs).

### 3.2. UV-VIS Spectroscopy

UV-VIS spectroscopy is one of the most used techniques for the primary characterization of metal NPs, synthesis monitorization, and long-term stability assessment of nanoparticle suspensions [48]. In general, metal NPs have unique optical properties, with very close valence and conduction bands in which electrons move freely. Upon excitation with external electromagnetic radiation, with a frequency equal to the oscillation frequency of these electrons, a characteristic UV-Vis absorption band occurs, known as *Localized Surface Plasmon Resonance* (LSPR) [49]. The characteristics of this band (e.g., position, height, full width at half maximum) are strongly dependent on the shape, average size, and concentration of the synthesized NPs [6,49].

**Figure 3:**
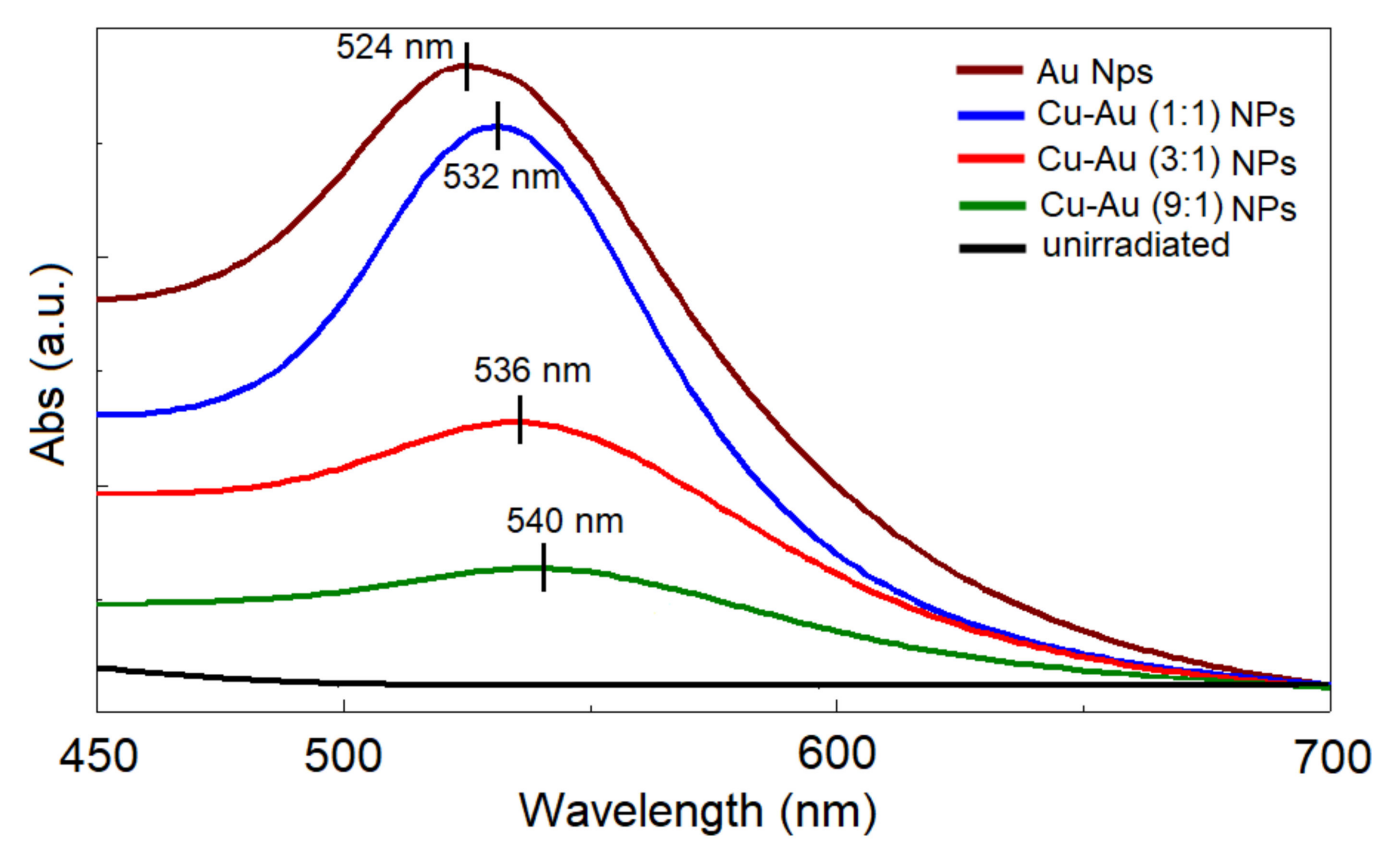
LSPR maxima recorded on radiochemically synthesized NPs.

The UV-Vis absorbance spectra recorded on the irradiated solutions (Figure 3) present a monoband structure in the wavelength range of 450–700 nm, with different LSPR characteristics as a function of copper concentration. This monoband structure, with absorption maxima between that of Au NPs (usually positioned at higher wavelengths than 520 nm [50]; 524 nm in our case) and those of Cu NPs (usually, between 550 and 650 nm [51]), is a confirmation of the formation of Cu-Au alloy nanoparticle structures through gamma radiation. The increase of Cu concentration leads to a red-shift of the absorption band, ranging from 532 nm (for Cu-Au (1:1) NPs) to 540 nm (for Cu-Au (9:1) NPs), accompanied by a decrease of the maximum absorption intensity.

With the increase in the copper content, a bathochromic shift is recorded, which can be associated with an increase in the particle dimensions [25]. The position of the UV-Vis peak represents a very good indicator regarding the particle dimensions. The 524 nm position of the pure gold NPs (0% copper) (Figure 3) suggests nanoparticle dimensions of around 20 nm [52].

**Figure 4:**
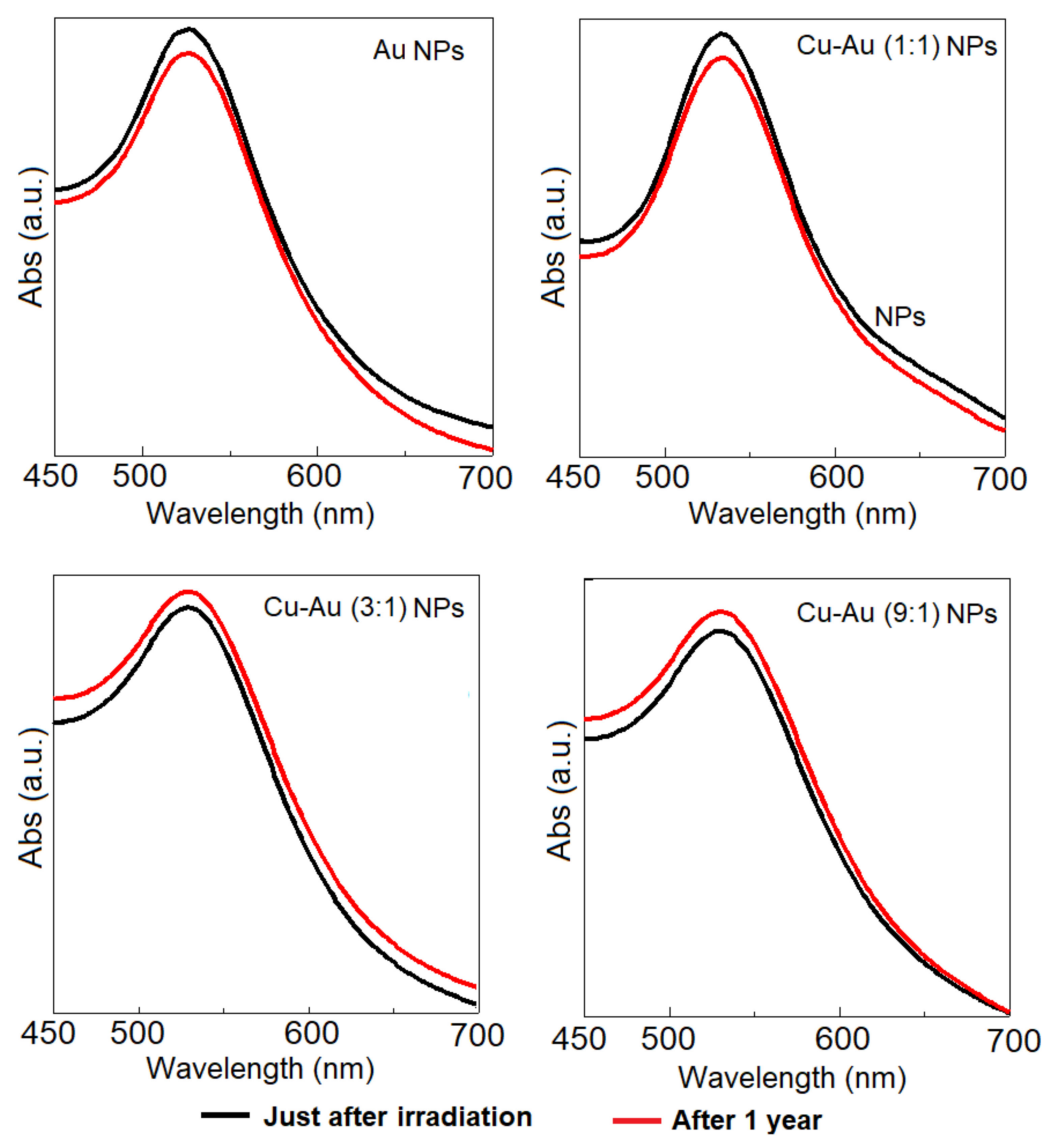
Stability of radiochemically synthesized Au NPs and Cu-au NPs.

In addition, UV-VIS spectroscopy is used for determining the stability of metal nanoparticle solutions immediately after synthesis and after long-term storage under defined conditions. These solutions show high stability during storage for 1 year, in laboratory conditions (i.e., r.t, alternance day/night), obviously because their UV-Vis absorption spectra remain practically unchanged for Au NPs and Cu-Au (1:1) NPs, and a slight absorbance increase for Cu-Au (3:1) NPs and Cu-Au (9:1) NPs (Figure 4).

It is worth mentioning that the NPs keep their stability at dilution with tap water for more than one year. The tap water dilution test is performed to provide an appropriate dilution method (antimicrobial efficiency tests will show that the solution is still effective at dilutions of over 75% for bacteria and over 50% for fungi and viruses).

### 3.3. FTIR Spectroscopy

**Figure 5:**
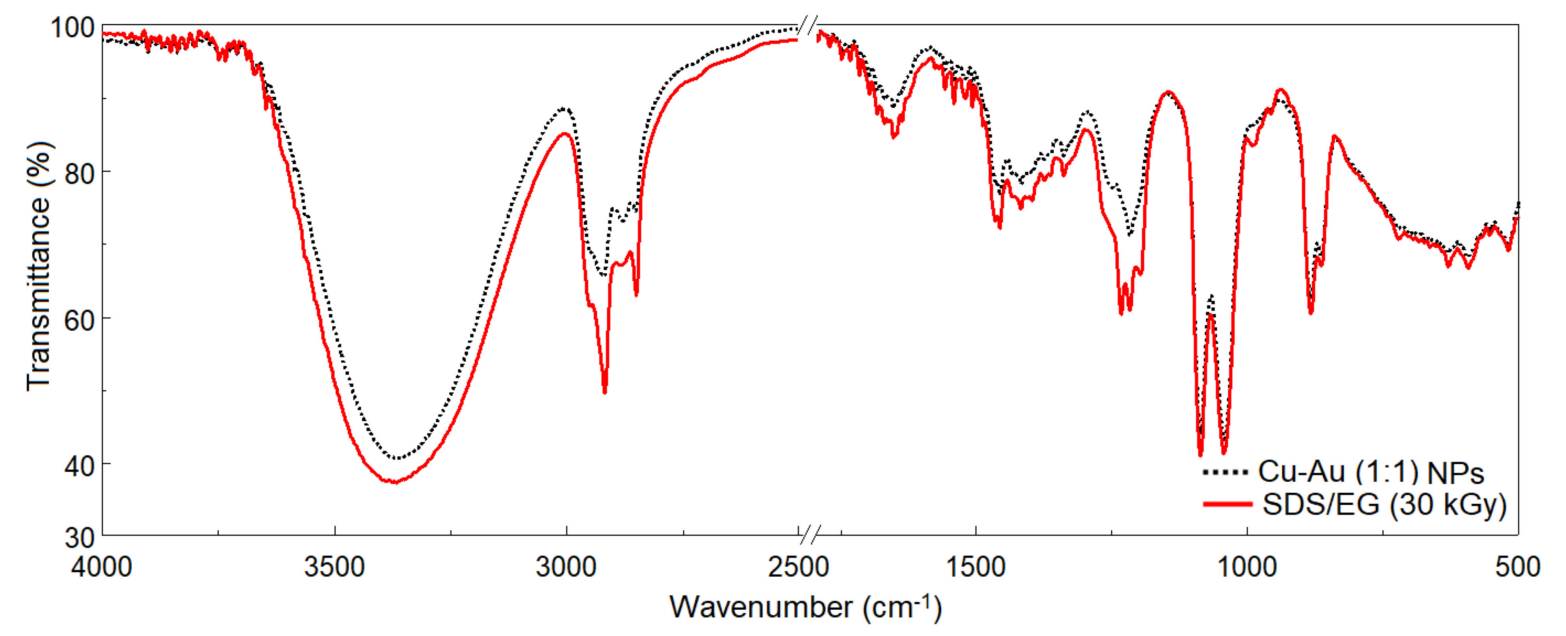
FTIR spectrum of Cu-Au (1:1) NPs compared to the irradiated SDS/EG system.

FTIR spectroscopy is employed to understand the role and mechanism of the coating agent (SDS) used in the synthesis of Cu-Au NPs. FTIR spectra (Figure 5) are dominated by the characteristic bands of SDS [53,54]: 3382 cm^−1^ (OH stretching vibration), 2951, 2920, 2850, 1646, and 1462 cm^−1^ (different FTIR vibration modes of C-H), 1231 cm^−1^ (S-O stretching vibration), 1075 cm^−1^ (C-C stretching vibration), and 827 and 579 cm^−1^ (asymmetric CH bending vibration).

The FTIR spectrum recorded on Cu-Au NPs coated with SDS shows certain spectral displacements: the OH vibration occurs at 3357 cm^−1^, S-O stretching vibration occurs at 1220 cm^−1^, and some bands change their intensity becoming smaller such as 2750–3000 cm^−1^ (CH_3_ symmetric and asymmetric stretching vibrations), 1582–1806 cm^−1^, and 1140–1300 cm^−1^. All these changes suggest the existence of powerful electrostatic interactions between the hydrophobic structure of the alkyl chain of SDS molecules and the surface of the Cu-Au alloy NPs. The stability in time of the UV signal corroborated with the detection in the IR spectrum of the SDS bands proves that the NPs are in the covered state.

### 3.4. XRD

**Figure 6:**
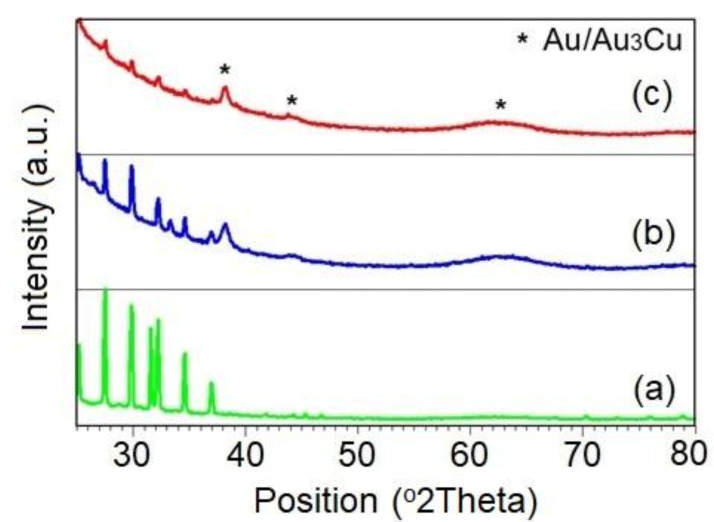
XRD patterns of SDS/EG system (**a**) and Cu-Au (1:1) NPs just after irradiation (**b**) and after 1 year (**c**).

Cu-Au NPs show the crystallographic phases of Au (PDF file #01-071-4616), cubic simple (space group Pm-3m (221)) and/or Au_3_Cu (PDF file #01-081-8007), and face centered cubic (space group Fd-3m (225)). The XRD pattern of the crystallized residue from the SDS/EG system is presented in Figure 6a to confirm the existence of some diffraction peaks from the matrix medium of the NPs; the matrix solution has been analyzed following the same preparation as presented previously. Qualitatively, after 1 year the NPs keep their stability, i.e., no occurrence of supplementary crystalline phase is noticed.

Table 1 shows the diffraction peaks’ main parameters (angular position, interplanar distance) for the crystallographic phases associated with the NPs, respectively, and the crystallite size is calculated according to the Debye–Scherrer relation:

(6) D=kλ/βcosθ

where *k* is a form factor (usually *k* = 0.9), *λ*—wavelength of the X-ray, *β*—full width at half maximum of the diffraction peak, *θ*—angular position.

Considering the theoretical elementary cell parameter (according to the ICDD PDF2 database) of the crystallographic phases of Au, respectively of Au_3_Cu, a slight difference in what concerns the experimental elementary cell parameter is observed. It can be explained by the existence of superficial stresses on the NPs (contribution of the reaction medium and the matrix medium in which they are immersed, imperfections (distortions) at the surface of the particles, etc.).

It should be noted that through the X-ray diffraction analysis the determination of the crystallite size does not always equate to the particle size. Theoretically, a particle can contain several crystallites (regions where the crystalline planes are arranged in parallel according to a given crystallographic direction), so the size of a particle should be larger than that of the crystallite. Experimentally, the characteristic peak, and its shape, sum up the contribution (distortions of the crystal lattice, interstitial impurities, surface tensions of NPs, etc.) of all crystallites oriented according to a certain crystallographic plane, so enough repetitive crystalline planes are needed to produce the diffraction phenomenon. Considering a uniform arrangement of NPs on the sample holder (as a result of exposing the solutions to eliminate the liquid matrix medium), but also the fact that the diffractogram sums the contribution of all these particles, the indication of the crystallite size calculated according to the Debye–Scherrer relation can be considered rather as a dimensional range in which their real dimension falls, so an average value of the particle size is as a qualitative indication and not purely quantitative.

### 3.5. TEM

**Figure 7:**
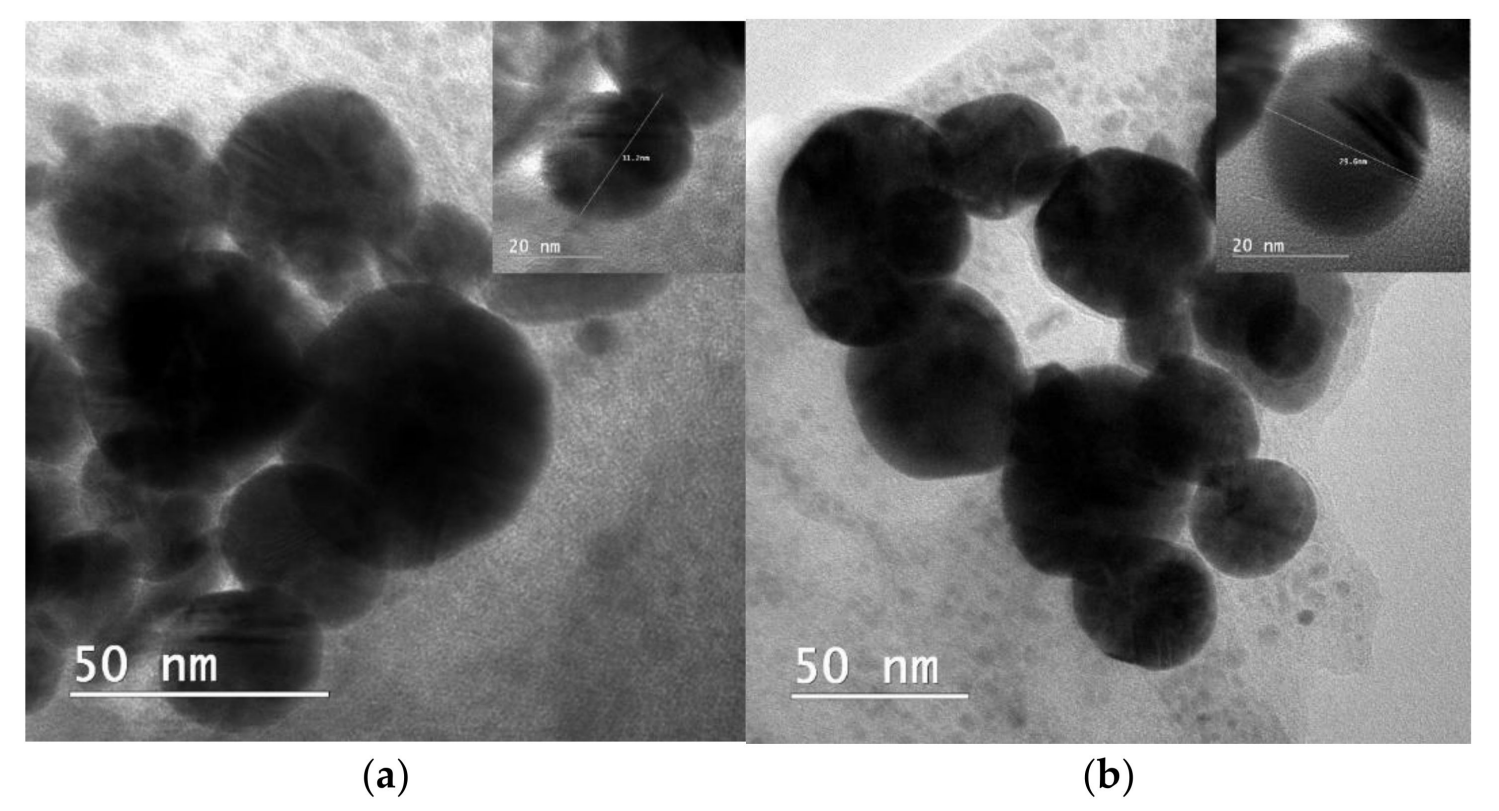
Representative TEM images of the obtained Cu-Au NPs (Cu-Au (1:1) NPs) just after irradiation (**a**) and after 1 year (**b**).

The transmission electron microscopic analysis confirms the presence of the different shape morphology (usually spherical) of the prepared Cu-Au NPs (Figure 7).

The diameter of the Cu-Au NPs range from 20 nm to 90 nm. No obvious differences in morphology are found between the initial sample and after 1 year storage, proving also the dimensional stability of Cu-Au NPs. However, there is a high tendency for the particle’s agglomeration. Generally, the dispersion and aggregation behavior of NPs is well controlled in the liquid phase by different mechanisms of surface particle interactions [55]. The FTIR analysis shows (Figure 5) that Cu-Au NPs are stabilized by some electrostatic interactions between the surface of the Cu-Au NPs and SDS. In addition, both the UV-Vis and ELS analyses also demonstrate the high stability over time of the nanoparticle solutions, with all these tests being performed in the liquid phase. Therefore, the tendency of particle agglomeration observed in TEM is explained by the fact that the TEM analysis is on an NPs solution deposited (by evaporation) on a solid support. By evaporation, the SDS passes into a crystalline state, and the stabilization mechanism of the NPs may be different from that in the liquid phase.

### 3.6. DLS and ELS

In DLS measurements, the results are calculated from a Cumulants analysis, in which a single particle size mode is assumed and a single exponential fit is applied to the autocorrelation function and the polydispersity describes the width of the assumed Gaussian distribution [56].

The hydrodynamic diameter (*d_H_*) that is equivalent to the effective diameter (d_eff_) is calculated from the autocorrelation function of the intensity of light scattered from particles with the assumption that the particles have a spherical form. It is the diameter of a compact sphere that diffuses at the same speed as the particle being measured, having the same translational diffusion coefficient as the particle being measured, defined via the Stokes–Einstein equation (Equation (7)) [57,58].

(7) $$d_{H}=\frac{k_{B}T}{3\pi \eta D_{}}$$

where *k_B_* is the Boltzmann constant, *T* is the absolute temperature, *η* is the viscosity of the liquid medium, and *D* is the translational diffusion coefficient. In the case of the synthesized Cu-Au NPs solution, deionized water is used that has the viscosity at 25 °C of 0.89 cP.

Although the fundamental size distribution generated by DLS is an intensity distribution, it is converted into a number distribution, using a multimodal size distribution (MSD) model to compare the results obtained by the TEM analysis.

The number distribution mean is calculated from the deconvolution of the measured intensity autocorrelation function of each Cu-Au NPs solution using a numerical algorithm and Mie theory, assuming all particles are spherical and homogeneous, and considering into calculation the real and imaginary components of the refractive index of Cu-Au NPs.

**Figure 8:**
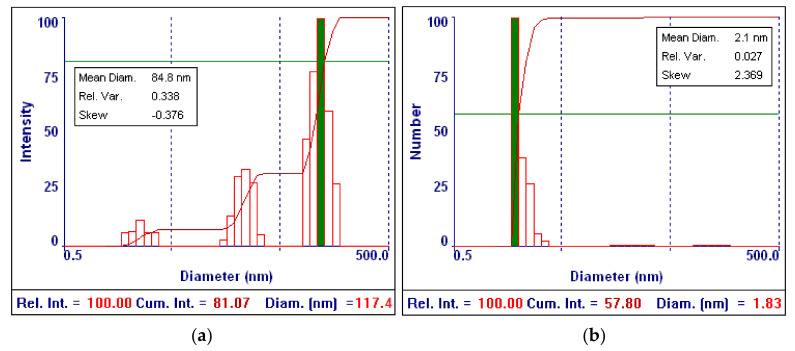
Particle size distribution of Cu-Au (1:1) NPs solution reported by intensity (**a**) and number (**b**).

Figure 8 shows the graphs of the particle size distribution by intensity, and number, respectively, obtained by the DLS measurements for the biologically relevant Cu-Au NPs solution (Cu-Au (1:1) NPs). Table 2 summarizes the results for all the synthesized Cu-Au NPs solutions.

The DLS measurements (Table 2 and Figure 8) reveal polydispersed particles with polydispersity values ranging around 0.22–0.33 and a small hydrodynamic diameter ranging around 60–92 nm, whereas the MSD mean diameter weighted by number varies around 1.8–3.3 nm.

The hydrodynamic diameter increases with the Cu content increase in the Cu-Au NPs solution. This finding is in agreement with the UV-Vis results, where from the recorded UV-Vis spectra (Figure 3) it is clear that the size of the Cu-Au NPs increases with the Cu content increase since the absorbance maxima are broadened and LSPR maxima are red-shifted.

The hydrodynamic diameter of Cu-Au NPs shows higher values than the MSD mean diameter weighted by number, which is close with the particle size determined by the TEM analysis (Figure 7). This is because the translational diffusion coefficient depends not only on the size of the particle “core”, but also on any surface structure, as well as the content and type of ions in the liquid medium [59]. The DLS analysis measures the hydrodynamic diameter, while in electron microscopy such as TEM the projected area of the particles is measured. The DLS analysis in general detects low amounts of large particles or clusters of smaller particles, while the TEM analysis usually reflects the size of primary particles and it is possible to not include larger clusters depending on sampling [60].

For all the Cu-Au NPs solutions, the number-weighted distribution shows a particle size distribution with the main population in a narrow range. All these distributions are right-skewed showing distortion from a symmetrical distribution with a steep rise on the left and long tail on the right side. In addition, the largest number of values is toward the left side of the distribution. The main population that is the most intense population contains a few groups of finer particles, while the other populations are in the form of a long tail with coarser particles. These particles can represent some aggregates or particles with different shapes than the spherical one that is considered in the Stokes–Einstein equation for the hydrodynamic diameter calculation. Since their relative percentage contribution of the size range is 0%, these populations can be neglected.

In ELS measurements with the 90Plus apparatus with BI-Zeta option, the velocity of Cu-Au NPs’ movement is measured under a current of 18.30–19.44 mA corresponding to an applied electric field of 15.37–15.78 V/cm by evaluating the frequency shift (Doppler shift) of scattered laser light relative to the reference beam (local oscillator) of 250 Hz. The signal strength is given by the magnitude of the scattered light seen by the detector (in kcps). The sample count rate is the signal scattered by the sample of the Cu-Au NPs solution, whereas the reference count rate is the signal from the reference beam. After the evaluation of the Doppler shift, the electrophoretic mobility (velocity per unit electric field) and zeta potential are determined (Table 3). Moreover, the optoelectronics of the 90Plus apparatus automatically allows for the detection of sign so that if the resultant Doppler shift is less than the frequency of the reference beam of 250 Hz, the sign of zeta potential is negative, as the obtained results confirm. The negative mobility of the charged Cu-Au NPs dispersed in an aqueous solution containing SDS/EG agents under the applied electric field means the surface of Cu-Au NPs is negatively charged.

The zeta potential is a function of the surface charge that develops when any material is placed in a liquid medium, and the functional groups on its surface will react with the surrounding liquid medium. As a result, the negative surface charge of Cu-Au NPs will attract the accumulation of positively charged ions existing in the liquid medium, and the counterions will form an electrostatic double layer composed by a Stern layer (ions strongly bound) and a diffused layer (ions loosely bound).

The magnitude of the final surface charges will depend on the strength of the acidic or basic groups of the surface or the amount of ions adsorbed to the surface of Cu-Au NPs and the pH of the colloidal solution. Thus, the zeta potential is given by the sum of the initial surface charge and the accumulated layer of ions. The potential at the surface of the electrostatic double layer (slipping plane) is defined as the zeta potential. Therefore, it is a very good index of the magnitude of the electrostatic repulsive interaction between particles [61].

**Figure 9:**
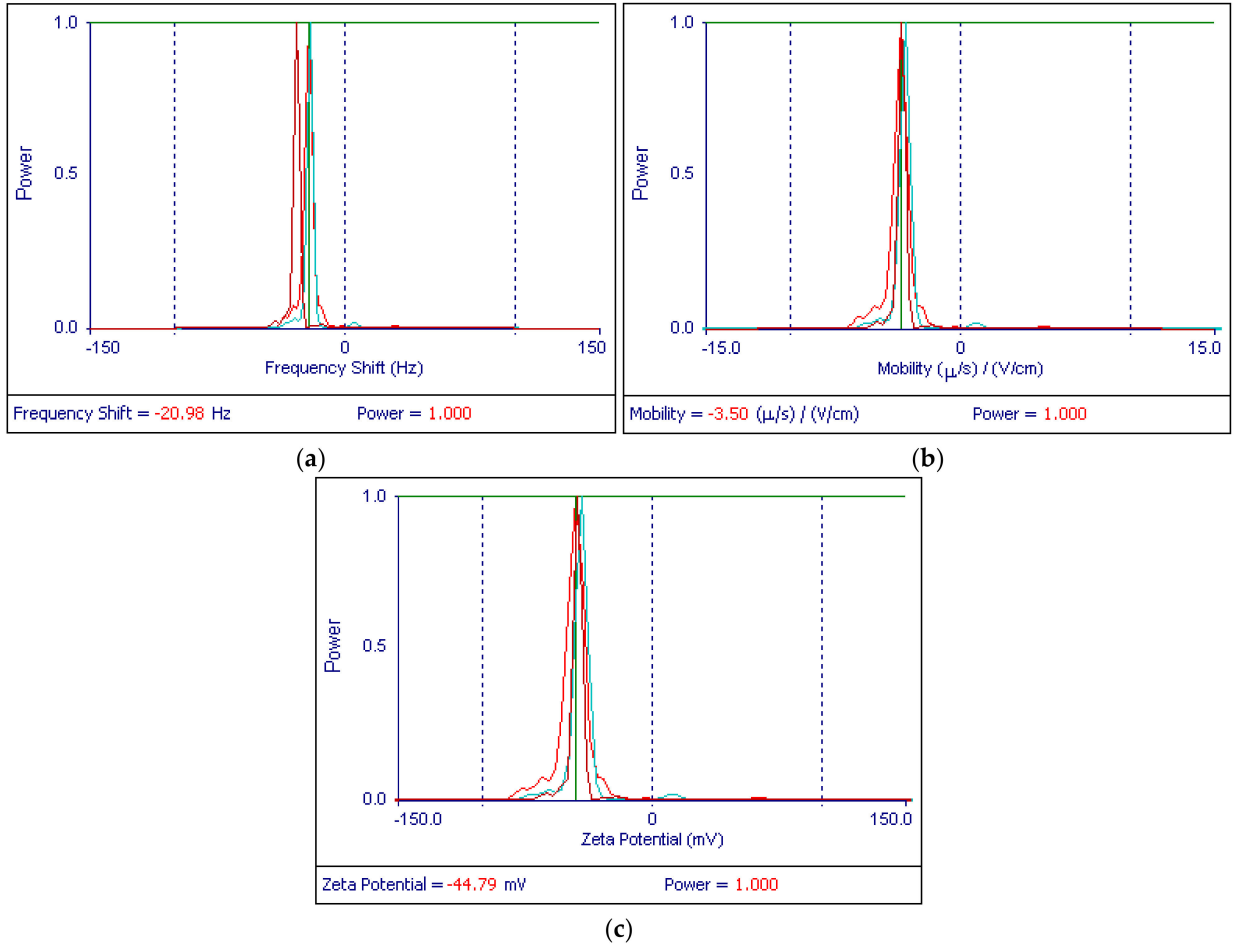
Comparing graphs of (**a**) frequency shift, (**b**) mobility, and (**c**) zeta potential of Cu-Au (1:1) NPs solution.

The zeta potential is often used to predict and control dispersion stability since the sign and magnitude of the zeta potential, as a function of pH, and content of salts, or dispersing and stabilizing agents are indicators of stability against flocculation or coagulation of particles [62]. The colloidal suspensions with zeta potentials more negative than −30 mV and more positive than +30 mV are considered stable because the particles have sufficient mutual repulsive forces to prevent the aggregation of particles leading to the dispersion stability [63]. The ELS measurements reveal that the Cu-Au (1:1) NPs from the colloidal solution have a negative zeta potential (Table 3 and in Figure 9) with values ranging between −41.49 ± 1.67 mV and −44.81 ± 2.07 mV that are more negative than -30 mV. As a result, Cu-Au (1:1) NPs are stable in the colloidal solution due to a strong electrostatic repulsion of their negatively charged surface. The obtained values of zeta potential showing a good stabilization of the Cu-Au NPs are probably the main reason in producing NPs with a narrow size distribution (as was disclosed from the DLS measurements) and a very low tendency towards aggregation.

### 3.7. Biocidal Activity

#### 3.7.1. Qualitative Assessments

As was already mentioned, the purpose of this paper was to develop a solution for the decontamination of surfaces based on Cu-Au alloy NPs. The preliminary microbiological tests showed a different bactericidal behavior, the most performant being the Cu-Au (1:1) NPs.

**Figure 10:**
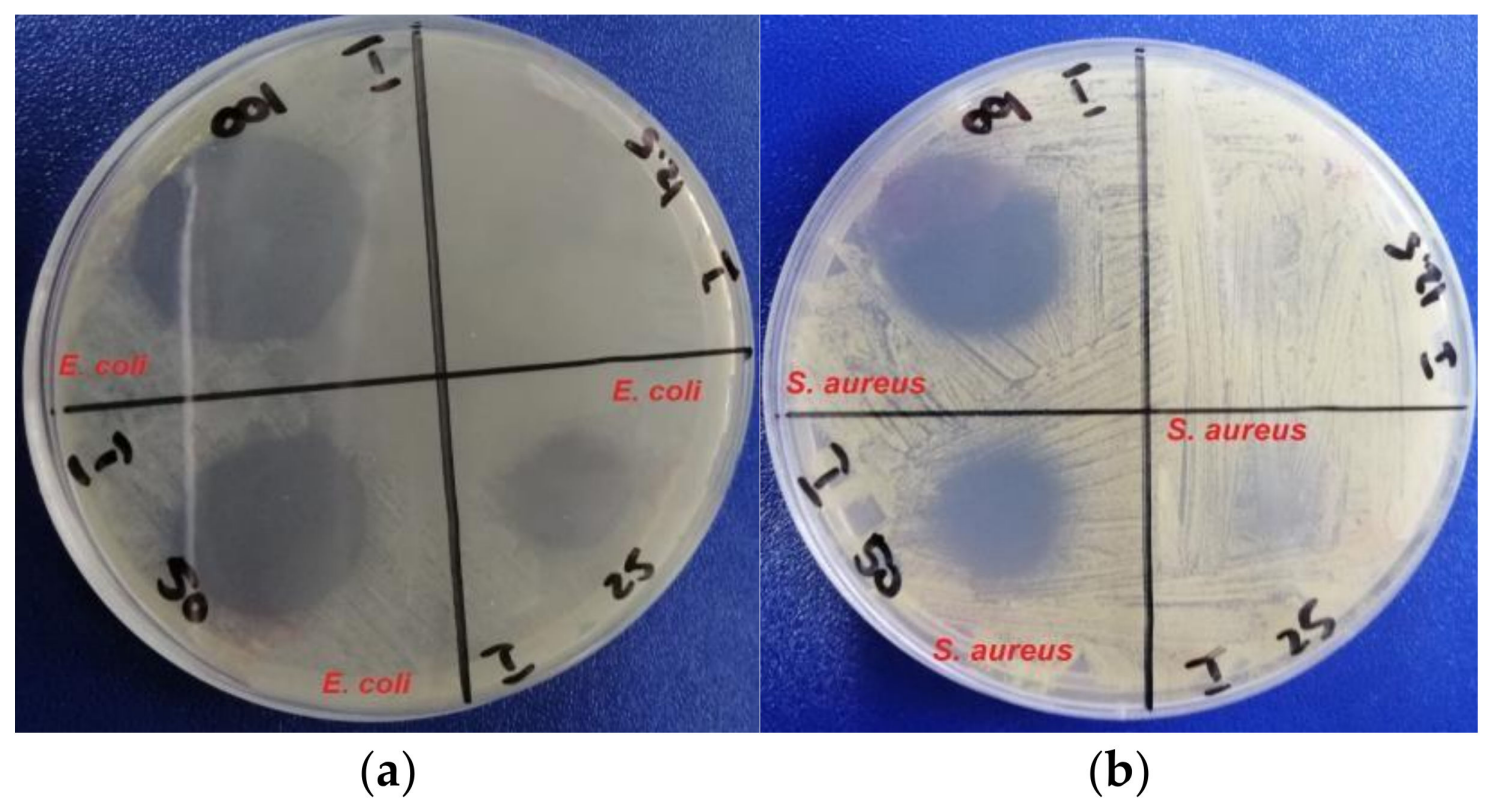
Typical antimicrobial efficiency assessment of Cu-Au (1:1) NPs against: (**a**) *Escherichia coli* ATCC 25922 and (**b**) *Staphylococcus aureus* ATCC 9737.

The tests were performed using an adapted disc diffusion [64] method on undiluted solution cu Cu-Au (1:1) NPs and diluted up to 12.5% with deionized water (Figure 10), against *Escherichia coli* ATCC 25922 and *Staphyloccocus aureus* ATCC 9737. The antibacterial efficiency was evaluated by measuring the growth diameter of inhibitions zones (Table 4).

The decrease in the inhibition zone of the Cu-Au Np solutions with the increase of the copper content is also a proof of the fact that the average dimensions of the NPs increase, according to the UV-Vis and DLS analyses, contributed to a decrease of the antimicrobial efficiency.

#### 3.7.2. Quantitative Assessments

##### Evaluation of the Bactericidal and Fungicidal Activity

**Figure 11:**
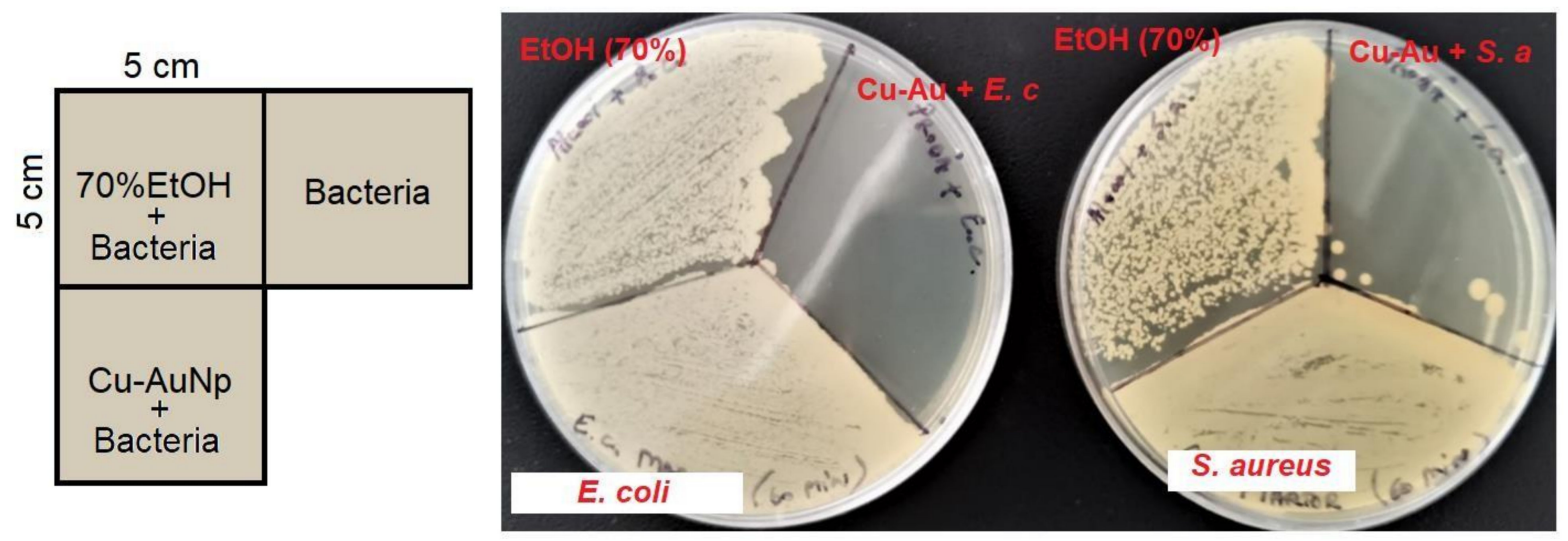
The surface decontamination efficiency of Cu-Au (1:1) NPs solution. Contact time: 1 h.

First, a quantitative experiment was carried out to simulate some real operating conditions, the antibacterial activity of Cu-Au (1:1) NPs being tested on a laboratory surface and compared with a solution of 70% ethanol (Figure 11).

The results show the high antibacterial efficiency of Cu-Au (1:1) NPs solution, much more efficient than the ethanol solution, traditionally used as the main component for most disinfectants [65]. This phenomenon can be explained by the fact that ethanol, a volatile compound, retains its antibacterial capacity only until its complete evaporation (i.e., 15–20 min). Thus, this time is not enough to reduce the entire bacterial load. In the case of Cu-Au (1:1) NPs, the bacterial load was reduced to a level where bacterial colonies for *S. aureus* can be counted (maximum six colonies/25 cm^2^), and for *E. coli* the elimination was almost complete (maximum one colony/25 cm^2^). The obtained results demonstrate the long-term antimicrobial efficacy of Cu-Au alloy NPs, proving, at the same time, their bacteriostatic properties.

As shown in Table 5, the Cu-Au (1:1) NPs solution proved to be bactericidal (decimal lg reduction ≥ 5) on three defined strains after 60 min of contact in dirty conditions (according to [39]). A significant difference was not observed between the results obtained for the tested bacterial strains. This could suggest that the solution is highly efficient for both types of bacteria, Gram-positive (*Staphylococcus aureus* and *Enterococcus hirae)* and Gram-negative (*Pseudomonas aeruginosa)*. The values obtained for the tested Cu-Au (1:1) NPs solution were almost similar with those obtained for other disinfectant formulations, such as chlorhexidine digluconate, isopropyl alcohol, hydrogen peroxide, etc. [66,67].

According to EN 13624 [40], the Cu-Au (1:1) NPs solution exerted a fungicidal activity after 60 min of contact at 20 °C in dirty conditions only against *Candida albicans* (Table 5). Similar results were previously described for aqueous solutions containing chlorhexidine digluconate [67]. The tested solution did not prove efficiency against *Aspergillus brasiliensis*. These results show that the Cu-Au (1:1) NPs solution had only yeasticidal activity and no fungicidal activity. This could be explained by the high tolerance exerted by *A. brasiliensis* to disinfectants, as it was previously reported that this strain was not inactivated by 50% ethanol [68] or other formulations (chlorhexidine digluconate, iodine, and hydrogen peroxide) [66].

##### Evaluation of the Virucidal Activity

Additionally, we evaluated the virucidal efficiency of the Cu-Au (1:1) NPs solution against various viruses (Poliovirus type 1, Adenovirus 5, Murine Norovirus, and Coronavirus 229E). The results obtained under dirty conditions at 80% solution concentration showed virucidal activity against Adenovirus 5, Coronavirus 229E, and Murine Norovirus when the requirements of the EN 14476 standard [41] were applied (Table 6). In contrast, the solution did not exert virucidal activity against Poliovirus type 1.

The differences observed in the virucidal activity between the tested strains could be related to the classification of the viruses according to their structure [69]. As previously reported, enveloped viruses (e.g., coronaviruses) are more susceptible to disinfectants than non-enveloped viruses, which are the most resistant to disinfectants due to their strong hydrophilic properties (e.g., poliovirus) [70]. In contrast, the non-enveloped viruses with reduced hydrophilic properties (e.g., adenoviruses, rotaviruses, and noroviruses) are slightly more sensitive to disinfectants [70]. The viruses that are the most susceptible to disinfectants are the enveloped viruses with a high lipid content, such as coronaviruses.

The virucidal effect was also validated for the diluted Cu-Au (1:1) NPs solution (50% *v/v*), only against Murine Norovirus and Coronavirus 229E, but not for Adenovirus and Poliovirus.

### 3.8. Evaluation of Human Cytotoxicity

Because toxicity can result from disinfectant residues leaching from improperly handling nanoparticle solutions, we assessed the cytotoxicity following standard reference methods used for that purpose [71]. These standards have been extensively used to evaluate new formulations that can contact human tissues and are particularly relevant for predicting in vivo toxic responses.

**Figure 12:**
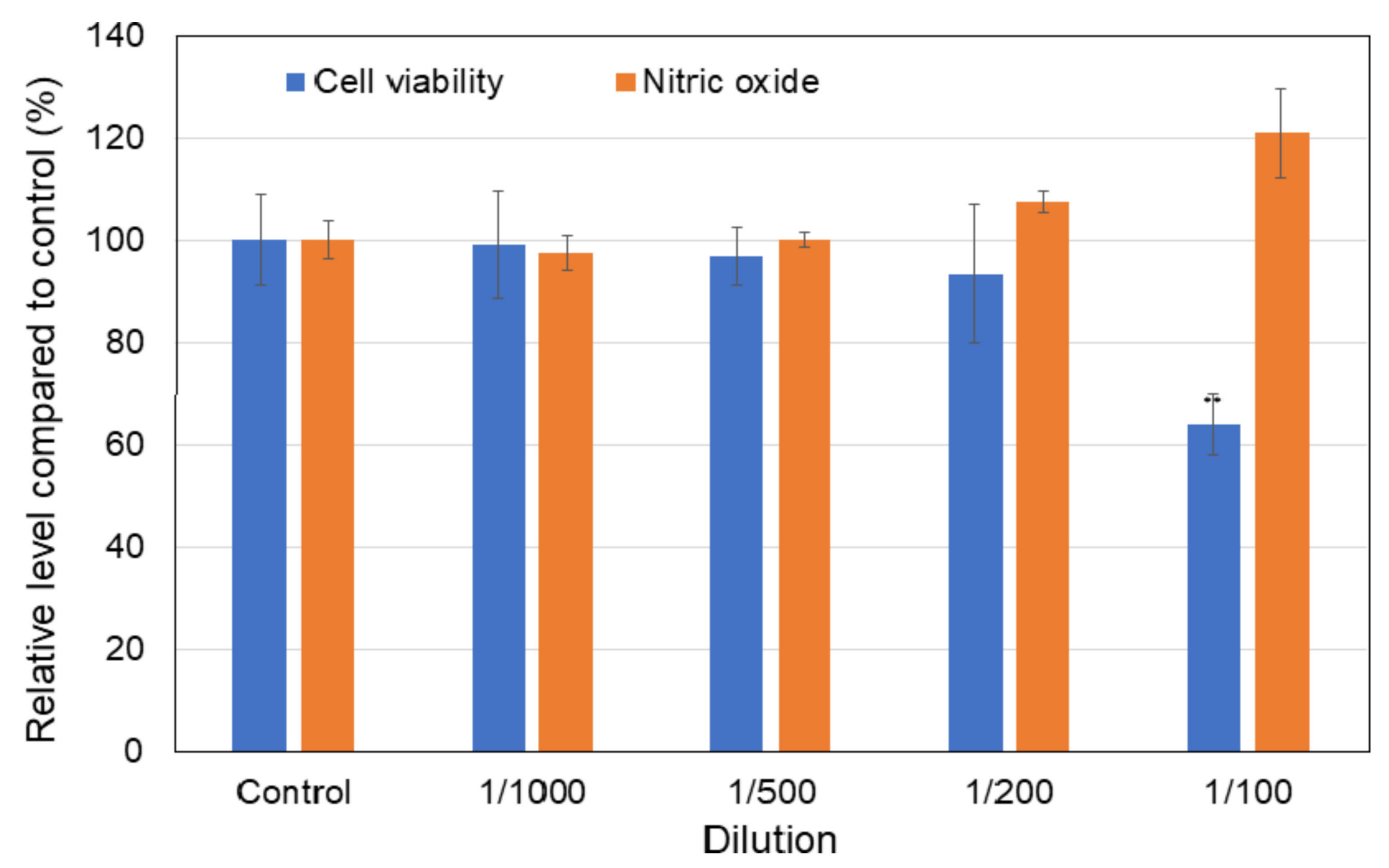
Cell viability results obtained by MTT assay and NO release measured by Griess assay after 6 h of cell growth in the presence of Cu-Au (1:1) NPs. Results are presented as mean ± standard deviation of three independent experiments (** *p* < 0.05, compared to control).

The effect on cell viability was studied by the MTT assay after an incubation period of 6 h. This time exposure was selected as an average daily time of working before washing. As shown in Figure 12, the dilutions of 1/1000, 1/500, and 1/200 did not significantly affect the number of viable cells after 6 h of incubation compared to the control.

The amount of NO released in the culture medium was assessed as a valuable indicator of inflammation and cell membrane damage after incubation with disinfectant solution. Figure 1 shows increases in NO release only after cell exposure to the dilution of 1/100, with the 1.2-fold increase of control correlating very well with the decrease by 37% for this dilution. These results prove that low quantities of the tested solution did not induce inflammation.

## 4. Conclusions

In this work, Cu-Au alloy NPs were obtained by gamma radiations, in a one-step process, in an aqueous solution, without the use of reducing agents.

The formation of NPs was confirmed both visually (by color change) and by different analysis techniques. Cu-Au nanoparticle solutions have high stability over time (>1 year), maximum absorption in UV-Vis between 524 and 540 nm (depending on the copper content), mostly spherical shapes, and dimensions between 20 and 90 nm.

Our study demonstrated the efficient bactericidal, fungicidal, and virucidal activity of Cu-Au nanoparticle solutions. Considering the solution’s biocidal efficiency, this could be used as a professional liquid or spray disinfectant on highly contaminated surfaces in medical units or other institutions. Besides this advantage, this radiochemically synthesized solution has mild toxicity in low doses on human skin fibroblasts, and the possible toxic responses in sensitized health care personnel have to be considered.

## 5. Patents

The results presented in this paper are the subject of a European patent application entitled “Copper-gold alloy nanoparticles and their manufacturing method”—PCT/RO2020/000017.

## Figures and Tables

**Table 1:** The diffraction main parameters of Cu-Au (1:1) NPs and ICDD PDF files of indexed crystalline phases.

Sample	Cryst. Phase	Cryst. Plane	Angular Position 2theta (°)	Interplanar Distance d (Å)	Cryst. Size D (nm)	Lattice Parameter a (Å)
Cu-Au (1:1) NPs just after irradiation	Au_3_Cu	(111)	38.113	2.35924	18.46	4.0863
Cu-Au (1:1) NPs after 1 year	Au_3_Cu		38.213	2.35329	17.92	4.0760
PDF file #01-071-4616	Au		38.175	2.35590	-	4.0876
PDF file #01-081-8007	Au_3_Cu		38.101	2.35998	-	4.0800

**Table 2:** Results obtained by DLS measurements and MSD mode for the synthesized Cu-Au NPs solutions.

Solution Type	Mean Hydrodynamic Diameter (*d_H_*) ± SD (nm)	Mean Polydispersity ± SD	MSD Mean Diameter Weighted by Number (nm)	MSD Diameter Weighted by Number for G(d) = 100% (nm)
Au NPs	66.9 ± 0.5	0.217 ± 0.011	3.5 ± 0.001	3.3
Cu-Au (1:1) NPs	59.7 ± 1.6	0.326 ± 0.008	2.1 ± 0.027	1.8
Cu-Au (3:1) NPs	73.0 ± 1.2	0.296 ± 0.013	2.1 ± 0.015	1.9
Cu-Au (9:1) NPs	92.0 ± 0.1	0.302 ± 0.011	2.3 ± 0.947	2.5

**Table 3:** Results obtained by ELS measurements for Cu-Au (1:1) NPs as a function of the measurement parameters.

Current (mA)	Electric Field (V/cm)	Conductance (µS)	Sample Count Rate (kcps)	Reference Count Rate (kcps)	Frequency Shift (Hz)	Electro-Phoretic Mobility (μs^−1^V^−1^cm)	Zeta Potential (mV)
19.44	15.37	3578	1817	8812	−21.96 ± 1.45	−3.47 ± 0.18	−44.39 ± 2.36
18.39	15.64	3385	1657	8579	−23.73 ± 1.65	−3.47 ± 0.08	−44.35 ± 1.00
18.30	15.78	3369	1714	8965	−22.91 ± 1.55	−3.50 ± 0.16	−44.81 ± 2.07

**Table 4:** Antimicrobial efficiency by diameter of the growth inhibition zone measurements.

Solution Type	Concentration (%)	Diameter of the Growth Inhibition Zone (mm)	
		*E. coli* ATCC 25922	*S. aureus* ATCC 9737
SDS/EG	-	8	15
Cu-Au (1:1) NPs	100	29	27
	50	23	19
	25	18	18
	12.5	-	-
Cu-Au (3:1) NPs	100	26	27
	50	23	22
	25	14	12
	12.5	-	-
Cu-Au (9:1) NPs	100	25	27
	50	19	21
	25	-	-
	12.5	-	-

**Table 5:** Bactericidal and fungicidal activity of pure (80% *v/v*) tested product in dirty conditions after a 60 min contact determined according to [39,40]. The results are an average of two replicates.

Test Organism	Decimal lg Reduction in Bacterial or Fungal Counts
*Staphylococcus aureus* ATCC6538	>5.13
*Pseudomonas aeruginosa* ATCC15442	>5.31
*Enterococcus hirae* ATCC10541	>5.11
*Candida albicans* ATCC10231	>4.10
*Aspergillus brasiliensis* ATCC16484	<1.37

**Table 6:** Virucidal activity of pure (80% *v/v*) and 50% *v/v* of the tested solution in dirty conditions after a 60 min contact determined according to [41] and expressed in units of TCID_50_/mL.

Test Organism	Decimal lg Reduction (TCID_50_/mL)	
Dilution of Cu-Au (1:1) NPs	80% *v/v*	50% *v/v*
Adenovirus 5 ATCC VR-5	>5.57	3.66
Coronavirus 229E ATCC VR-740	>4.99	4.49
Murine Norovirus strain S99 Berlin	>5.91	4.08
Poliovirus type 1 ATCC VR-192	3.00	1.74

## Data Availability

Data are available upon request from the corresponding author.
